# Quantitative target analysis and kinetic profiling of acyl-CoAs reveal the rate-limiting step in cyanobacterial 1-butanol production

**DOI:** 10.1007/s11306-015-0940-2

**Published:** 2016-01-04

**Authors:** Shingo Noguchi, Sastia P. Putri, Ethan I. Lan, Walter A. Laviña, Yudai Dempo, Takeshi Bamba, James C. Liao, Eiichiro Fukusaki

**Affiliations:** Department of Biotechnology, Graduate School of Engineering, Osaka University, 2-1Yamadaoka, Suita, Osaka 565-0871 Japan; Department of Chemical and Biomolecular Engineering, University of California, Los Angeles, 5531 Boelter Hall, 420 Westwood Plaza, Los Angeles, CA 90095 USA

**Keywords:** Cyanobacteria, 1-Butanol, Acyl-CoAs, Quantitative target analysis, Kinetic profiling, Liquid chromatography-mass spectrometry (LC–MS)

## Abstract

Cyanobacterial 1-butanol production is an important model system for direct conversion of CO_2_ to fuels and chemicals. Metabolically-engineered cyanobacteria introduced with a heterologous Coenzyme A (CoA)-dependent pathway modified from *Clostridium* species can convert atmospheric CO_2_ into 1-butanol. Efforts to optimize the 1-butanol pathway in *Synechococcus elongatus* PCC 7942 have focused on the improvement of the CoA-dependent pathway thus, probing the in vivo metabolic state of the CoA-dependent pathway is essential for identifying its limiting steps. In this study, we performed quantitative target analysis and kinetic profiling of acyl-CoAs in the CoA-dependent pathway by reversed phase ion-pair liquid chromatography-triple quadrupole mass spectrometry. Using ^13^C-labelled cyanobacterial cell extract as internal standard, measurement of the intracellular concentration of acyl-CoAs revealed that the reductive reaction of butanoyl-CoA to butanal is a possible rate-limiting step. In addition, improvement of the butanoyl-CoA to butanal reaction resulted in an increased rate of acetyl-CoA synthesis by possibly compensating for the limitation of free CoA species. We inferred that the efficient recycling of free CoA played a key role in enhancing the conversion of pyruvate to acetyl-CoA.

**Electronic supplementary material:**

The online version of this article (doi:10.1007/s11306-015-0940-2) contains supplementary material, which is available to authorized users.

## Introduction

Cyanobacterial biofuel production is considered as an attractive approach for providing sustainable energy resources due to its capability to utilize solar energy and CO_2_ as sole energy and carbon sources, respectively (Machado and Atsumi 2012; Jin et al. 2014). So far, production of various biofuels such as ethanol (Gao et al. 2012; Deng and Coleman 1999; Dexter and Fu 2009), 1-butanol (Lan and Liao 2011, 2012; Lan et al. 2013), isobutanol (Atsumi et al. 2009; Varman et al. 2012), 2,3-butanediol (Oliver et al. 2013), fatty acid (Liua et al. 2011), alkanes (Wang et al. 2013) and isoprene (Lindberg et al. 2010) have been achieved via engineered bioprocess in cyanobacteria.

1-Butanol is a promising gasoline replacement compared to the more commonly used ethanol due to several advantages. Specifically, 1-butanol is less corrosive and has a higher energy density than ethanol (Dürre 2007). Previously, we were able to successfully engineer the cyanobacteria *Synechococcus elongates* PCC 7942 to produce 1-butanol under anoxic and dark conditions via a modified CoA-dependent pathway (Lan and Liao 2011) based on the acetone-butanol-ethanol fermentation in *Clostridium* species (Gheshlaghi et al. 2009). In a stepwise process, we enabled cyanobacterial 1-butanol production under photosynthetic condition by introducing ATP as an alternative driving force for acetoacetyl-CoA synthesis, which is the first committed step in the 1-butanol pathway (Lan and Liao 2012). Furthermore, 1-butanol productivity was dramatically improved under photosynthetic condition by substituting the oxygen-sensitive butanal dehydrogenase with an oxygen-tolerant CoA-acylating propionaldehyde dehydrogenase (Lan et al. 2013). However, the maximum titer in cyanobacteria is still low compared to the 1-butanol-producing *Escherichia coli* with the heterologous CoA-dependent pathway; the 1-butanol titer is 317 mg/L from CO_2_ in 12 days using *Synechococcus elongatus* and 30 g/L from glucose in 7 days using *Escherichia coli* (Lan et al. 2013; Shen et al. 2011). Although the improvement of 1-butanol productivity in cyanobacteria has been made by focusing on the CoA-dependent 1-butanol biosynthesis pathway, there is no information about in vivo metabolic state of the CoA-dependent pathway in cyanobacteria. Therefore, intracellular metabolic profiling of the intermediates in the CoA-dependent pathway is expected to be essential for gaining clues for further optimization of the 1-butanol biosynthesis pathway.

To the best of our knowledge, there is no report on the investigation of the in vivo metabolic state of 1-butanol-producing cyanobacteria in spite of the attention given to photosynthetic 1-butanol production system. To tackle this problem, we performed targeted quantitative analysis and kinetic profiling of acyl-CoAs in the modified CoA-dependent pathway by using reversed phase ion-pair liquid chromatography-triple quadrupole mass spectrometry (RP-IP-LC)/QqQ-MS. This study aimed to gain insights into further CoA-dependent pathway optimization for the improvement of 1-butanol productivity in *Synechococcus elongatus*. Our results validated at the metabolite level that ATP-driven malonyl-CoA-mediated pathway functioned as intended and indicated a possible rate-limiting reaction in the pathway. Moreover, this study revealed an unexpected increase in the acetyl-CoA synthesis rate due to the introduction of the oxygen-tolerant aldehyde dehydrogenase and explained the possible reason for the enhanced conversion of pyruvate to acetyl-CoA. The results of this study will be helpful in improving productivity of 1-butanol and other chemicals in cyanobacteria via the modification of the CoA-dependent pathway.

## Materials and methods

### Cyanobacterial strains, plasmids and oligonucleotides used

*Synechococcus elongatus* strains used in this study are listed in Table 1. Plasmid pEL256 was constructed by excluding genes *pduP* and *yqhD* from plasmid pSR3 (Lan et al. 2013). Using pSR3 as template, fragments 1 and 2 were amplified by PCR with primer pairs EL-pEL256-P1-F/EL-pEL256-P1-R and EL-pEL256-P1-F/ EL-pEL256-P2-R, respectively. The two fragments were then ligated using the Gibson isothermal DNA assembly method (Gibson et al. 2009). Strain BUOH-SE w/o *pduP* was constructed by transforming strain EL9 with plasmid pEL256.

**Table 1:** Strains, plasmids and oligonucleotides used

Strains	Important Genotype	Reported 1-butanol production (mg/L)	Source
PCC 7942	Wild-type *Synechococcus elongatus* PCC 7942		
EL9	P_Trc_::His-tagged *T. denticola ter* integrated at NSI in PCC 7942 genome		Lan and Liao 2011
EL14	P_Trc_:: His-tagged *T. denticola ter* integrated at NSI and P_LlacO1_:: *atoB*, *adhE2*,*crt*, *hbd* integrated at NSII in PCC 7942 genome	<2	Lan and Liao 2011, 2012
EL20	P_Trc_:: His-tagged *T. denticola ter* integrated at NSI and P_LlacO1_:: *nphT7, adhE2, crt, hbd* integrated at NSII in PCC 7942 genome	6.4	Lan and Liao 2012
EL22	P_Trc_:: His-tagged *T. denticola ter* integrated at NSI and P_LlacO1_:: *nphT7, bldh, yqhD, phaJ, phaB* integrated at NSII in PCC 7942 genome	29.9	Lan and Liao 2012
BUOH-SE	P_Trc_:: His-tagged *T. denticola ter* integrated at NSI and P_LlacO1_:: *nphT7, pduP_S. ent*, *yqhD, phaJ, phaB* integrated at NSII	317	Lan et al. 2013
BUOH-SE w/o *pduP*	P_Trc_:: His-tagged *T. denticola ter* integrated at NSI and P_LlacO1_:: *nphT7, phaJ, phaB* integrated at NSII		This study

Plasmids	Genotype	Reported 1-butanol production (mg/L)	Source
pSR3	Kan^R^; NSII targeting; P_LlacO1_:: *nphT7, pduP_S. ent, phaJ, phaB*		Lan et al. 2013
pEL256	Kan^R^; NSII targeting; P_LlacO1_:: *nphT7, phaJ, phaB*		This study

Primers	Sequences (5′ → 3′)	Reported 1-butanol production (mg/L)	Source
EL-pEL256-P1-F	AGGAGATATACCATGTCTGCGCAATCTCTC		This study
EL-pEL256-P1-R	CTAGATCTCGCAGCGTAAAGCCG		This study
EL-pEL256-P1-F	CGGCTTTACGCTGCGAGATCTAG		This study
EL-pEL256-P2-R	CATGGTATATCTCCTTTACCACTCGATCAGC		This study

Oligonucletide primer sequence is from 5′ to 3′. Reported 1-butanol production values are according to the corresponding source publications. For EL14, EL20 and EL22 each data was taken at 18 days since IPTG induction under light and aerobic condition (Lan and Liao 2012). For BUOH-SE the data was taken at 12 days since IPTG induction under light and aerobic condition (Lan et al. 2013)

*Ter* (*T. denticola*) trans-2-enoyl-CoA reductase, *atoB* (*E. coli*) acetyl-CoA acetyltransferase, *adhE* (*C. acetobutylicum*) bifunctional aldehyde/alcohol dehydrogenase, *crrt* (*C. acetobutylicum*) *hbd* (*C. acetobutylicum*), *nphT7* (*Streptomyces* sp. Strain CL190) acetoacetyl-CoA synthase, *bldh* (*C. saccharoperbutylacetonicum*) CoA-acylating butanal dehydrogenase, *yqhD* (*E. coli*) NADPH-dependent alcohol dehydrogenase, *phaJ* (*A. caviae*) (*R*)-specific crotonase, *phaB* (*R. eutropha*) acetoacetyl-CoA reductase, *pduP* (*S. enterica*) CoA-acylating propionaldehyde dehydrogenase, *Kan* ^*R*^ kanamycin resistance

### Culture medium and growth conditions

The culture medium and conditions used in this study were based on Lan et al. (2013) with minor modifications. All cyanobacterial strains were grown on modified BG-11 plates (1.5 g/L NaNO_3_, 0.0272 g/L CaCl_2_·2H_2_O, 0.012 g/L ferric ammonium citrate, 0.001 g/L Na_2_EDTA, 0.040 g/L K_2_HPO_4_, 0.0361 g/L MgSO_4_·7H_2_O, 0.020 g/L Na_2_CO_3_, ×1000 trace mineral, 1.43 g H_3_BO_3_, 0.905 g MnCl_2_·4H_2_O, 0.111 g ZnSO_4_·7H_2_O, 0.195 g Na_2_MoO_4_·2H_2_O, 0.0395 g CuSO_4_·5H_2_O, 0.0245 g Co(NO_3_)_2_·6H_2_O, 0.00882 g/L sodium citrate dihydrate) agar (1.5 % w/v). The strains were cultured in 50 mL of BG-11 medium containing 50 mM NaHCO_3_ and 20 mM HEPES–KOH (pH 7.5) in 300 mL screw cap flasks. In the case of mutants, 20 mg/L spectinomycin and 10 mg/L kanamycin were added. Three different flasks were prepared for each strain as biological replicates. Cultures were grown with continuous shaking at 30 °C under 50 μmol/s/m^2^ light condition. The initial cell density of culture was set at OD_730_ = 0.04. Induction of the growing culture with IPTG (1 mM final concentration) was done at cell density OD_730_ equal to 0.3–0.4. The pH was adjusted to eight everyday using 10 M HCl. After 1 day of IPTG induction, 5 mL of the culture was removed from the flask and 5 mL of fresh BG-11 containing 500 mM NaHCO_3_ and IPTG were added back to the culture.

### Sampling and extraction procedure

Cells were harvested by fast filtration using 0.2 μm pore size Omnipore filter disks (Millipore, USA) and subsequently washed with pre-cooled 70 mM ammonium bicarbonate. The filter papers containing the cells were immediately placed in an aluminum cylinder pre-chilled with liquid nitrogen for immediate quenching of cyanobacterial metabolic activity. The quenching procedure was performed in less than 30 s. The filter papers were stored in 15 mL centrifuge tubes at −80 °C until extraction.

For quantitative target analysis, culture broth equivalent to 5 mg dry cell weight was harvested after 3 days of IPTG induction. Intracellular metabolites were extracted using 2 mL of pre-chilled 80 % methanol with 100 μL of ^13^C-internal standard followed by vortexing for 30 s and sonication for 20 s. After precipitating the cells by centrifugation at 10,000×*g* for 5 min at 4 °C, two separate 2 mL microfuge tubes were each dispensed with 800 µL of the resulting supernatant. Subsequently, 640 μL of chloroform and 480 μL of ultrapure water were added into each tube before vortexing for 30 s. The samples were centrifuged at 16,000×*g* for 5 min at 4 °C and 800 μL of the resulting polar phase was collected and filtered using a 0.20 μm Millex-LG filter (Millipore, USA). The filtered supernatant was concentrated using a centrifugal concentrator (VC-36S, TAITEC, Japan) for 2 h. Identical samples were combined and subsequently freeze dried.

### Absolute quantification of intracellular metabolites

The intracellular concentration of targeted metabolites namely acetyl-CoA, malonyl-CoA, butanoyl-CoA and pyruvate was quantified using ^13^C-labeled cell extract as internal standard (Wu et al. 2005; Dempo et al. 2014; Bennett et al. 2008). To prepare the cell extracts, wild type and BUOH-SE strains were cultured in 20 mL of BG-11 containing 50 mM NaH^13^CO_3_ (>98 at.% ^13^C, Isotec Inc., USA) for 2 days. Subsequently, the cells were inoculated in 100 mL of BG-11 containing 50 mM NaH^13^CO_3_ at an initial OD_730_ of 0.04. For medium feeding, BG-11 with 500 mM NaH^13^CO_3_ was added into the culture. After 3 days of IPTG induction, cells were harvested by fast filtration using 1 μm pore size Omnipore filter disks (Millipore, USA) and subsequently washed with pre-cooled deionized water. The intracellular metabolites extraction was performed using the method described above and repeated four times excluding the freeze drying step. After centrifugal concentration for 2 h, all the samples were pooled in a 15 mL centrifuge tube. This pooled extract was used as an internal standard. The calibration curves for each metabolite were constructed according to Dempo et al. (2014) with minor modifications. Predetermined amounts of unlabeled standard mixtures were added into 2 mL of 80 % methanol with ^13^C-labelled internal standard and extracted using the same procedure as described above. The peak area ratios of U-^12^C to U-^13^C metabolites were calculated using the LabSolutions software (Shimadzu Co., Japan) and plotted against their corresponding predetermined amounts of authentic standards. The calibration curves of targeted metabolites are listed in Table S1.

### ^13^C-labelling experiment

For metabolic turnover analysis, ^13^C-labelling experiment was carried out according to Hasunuma et al. (2013) with minor modifications. Cells pre-cultivated for 3 days after IPTG induction using the conditions mentioned above were filtered and resuspended in 50 mL of BG-11 with 25 mM NaH^13^CO_3_. Four mL of culture broth was filtered at each time point (0.5, 1, 2, 5, 10 and 30 min) and the cells that were retained on the filter papers were processed as described in the previous section. Based on Hasunuma et al. (2010), the ^13^C fraction was calculated using the equation:

$$ m_{i} (\% ) = \frac{{M_{i} }}{{\mathop \sum \nolimits_{j = 0}^{n} M_{j} }} \times 100 $$

$$ ^{ 1 3} {\text{C fraction }}\left( \% \right) = \mathop \sum \limits_{i = 1}^{n} \frac{{i \times m_{i} }}{n} $$

where $M_{i}$ represents the peak area for a given metabolite with $i$^13^C atoms, $m_{i}$ represents relative peak area for each isotopomer and $n$ represents the number of carbon atoms in the metabolite. Metabolic turnover rate of each metabolite was calculated from the initial slope of the ^13^C fraction change with respect to time (0.5–5 min).

### IP-LC/QqQ-MS analysis

The freeze-dried samples were dissolved in 30 μL of ultrapure water for IP-LC/QqQ-MS analysis using a Shimadzu Nexera UHPLC system coupled with LCMS 8030 plus (Shimadzu Co., Japan). The protocol for LC/QqQ-MS analysis was based on Dempo et al. (2014) using an L-column 2 ODS (150 mm × 2.1 mm, 3 μm, Chemicals Evaluation and Research Institute, Japan). The mobile phase A used was 10 mM tributylamine and 15 mM acetic acid in water, while mobile phase B was methanol. The flow rate was 0.2 mL/min and column oven temperature was 40 °C. Gradient curves were as follows: for acyl-CoAs, 0 % B at 0, 50 % B at 1, 60 % B at 10, 90 % B at 11–13, 0 % B at 14-20 min, and for pyruvate, 0 % B at 0, 15 % B at 2, 25 % B at 7, 50 % B at 9, 100 % B at 11–13, 0 % B at 13.01–18 min. The analysis mode was negative ion mode. Probe position was +1.5 mm, desolvation line temperature was 250 °C, nebulizer gas flow was 2 L/min, drying gas flow was 15 L/min, and heat block temperature was 400 °C. The IP-LC/QqQ-MS analysis was performed with multiple reaction monitoring (MRM). Analytical parameters for the quantitative analysis and turnover analysis are listed in Table S2.

### Determination of intracellular NADH and NAD^+^ concentration

Fluorescent NAD/NADH detection kit (Cell Technology, USA) was used for quantification of intracellular NADH and NAD^+^ concentration following the manufacturer’s protocol. For each extraction, 1 mL of culture broth (3 days after IPTG induction) was collected in a 1.5 mL microfuge tube and subsequently centrifuged at 16,000×*g* for 1 min at 4 °C. After removal of the supernatant, the resulting cell pellet was immediately immersed in liquid nitrogen. Next, the pellet was resuspended in 100 μL of the NAD^+^ or NAD(H) extraction buffer and 100 μL of the NAD/NADH lysis buffer. After the samples were heated at 60 °C for 20 min, 100 μL of the reaction buffer and 200 μL of the opposite (NAD(H) or NAD^+^) extraction buffer were added into the tube. The lysate was mixed and centrifuged at 8,000 g for 5 min at 4 °C. The supernatant was then retrieved for NAD^+^ and NADH analysis. The supernatant was added with the enzyme mix and fluorescent NADH reaction reagent according to the manufacturer’s instructions. The mixture was incubated at room temperature under a dark condition for 90 min. Readings were taken using an ARVO MX 1420 multilabel counter (PerkinElmer) with excitation at 544 nm and emission at 590 nm.

## Results

### Comparison of two pathways for the formation of acetoacetyl-CoA from acetyl-CoA using targeted CoA profiling method

**Fig. 1:**
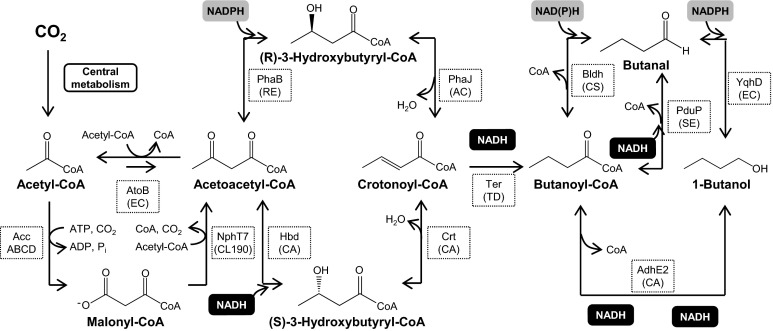
Schematic representation of CoA-dependent 1-butanol biosynthesis pathway. Abbreviations: *AccABCD* acetyl-CoA carboxylase, *NphT7* acetoacetyl-CoA synthase, *AtoB* acetyl-CoA acetyltransferase, *Hbd* 3-hydroxybutyryl-CoA dehydrogenase, *Crt* crotonase, *PhaB* acetoacetyl-CoA reductase, *PhaJ* (*R*)-specific crotonase, *Ter* trans- enoyl-CoA reductase, *AdhE2* bifunctional aldehyde/alcohol dehydrogenase, *Bldh* CoA-acylating butanal dehydrogenase, *PduP* CoA-acylating propionaldehyde dehydrogenase, *YqhD* NADPH-dependent alcohol dehydrogenase, *EC* *Escherichia coli*, *CL190* *Streptomyces* sp. *Strain CL190*, *CA* *Clostridium acetobutylicum*, *RE* *Ralstonia eutropha*, *AC* *Aeromonas caviae*, *TC* *Treponema denticola*, *CS* *Clostridium saccharoperbutylacetonicum* *N1-4*, *SE* *Salmonella enterica*

**Fig. 2:**
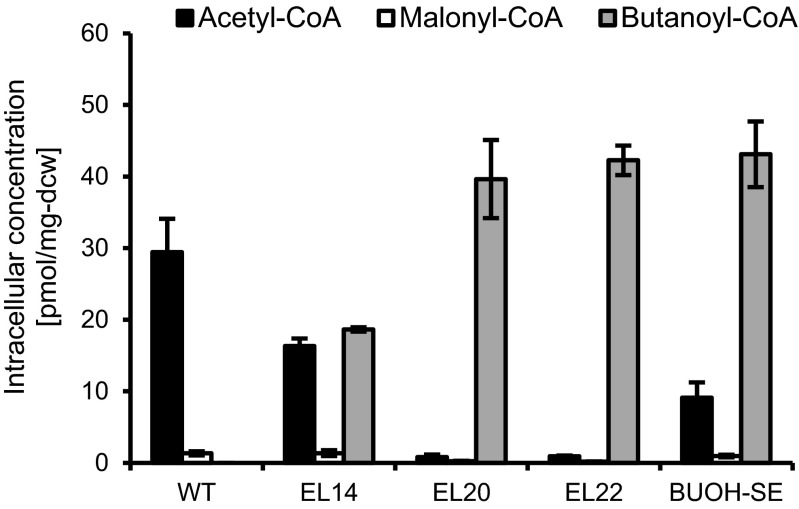
Intracellular concentration of targeted acyl-CoAs namely acetyl-CoA, malonyl-CoA and butanoyl-CoA in strain wild type, EL14, EL20, EL22 and BUOH-SE. The concentration is described using pmol/mg-dry cell weight (dcw) as a unit. Error bar shows standard deviation. 1-butanol concentration of all strains at 3 days after IPTG induction was lower than the LLOQ of GC-FID (lower limit of quantification: 5 mg/L), with the exception of BUOH-SE strain (86 mg/L)

Formation of acetoacetyl-CoA from acetyl-CoA in CoA-dependent 1-butanol biosynthesis can be achieved via two pathways. One is via the direct condensation of bimolecular acetyl-CoA catalyzed by thiolase (*atoB*-encoded enzyme) present in strain EL14 (Fig. 1; Table 1). This condensation reaction is thermodynamically unfavorable since it requires an acetyl-CoA pool to drive the reaction forward, which was thought to be unsuitable for the effective conversion of acetyl-CoA to acetoacetyl-CoA under photosynthetic condition (Lan and Liao 2011). The other is an ATP-driven malonyl-CoA-mediated reaction catalyzed by acetyl-CoA carboxylase and acetoacetyl-CoA synthase (*accABCD* and *nphT7*-encoded enzyme, respectively) present in strains EL20, EL22 and BUOH-SE (Fig. 1; Table 1). ATP, serving as an alternative driving force, enabled 1-butanol biosynthesis under photosynthetic condition (Lan and Liao 2012). To further optimize the pathway, it is important to validate the proposed ATP-dependent function at the metabolite level. To address this, we performed quantitative metabolic profiling of acyl-CoAs in the 1-butanol biosynthesis pathway. Compared to the wild type, the intracellular concentration of acetyl-CoA in strain EL14 decreased by 1.8-fold (Fig. 2) while the concentration of malonyl-CoA did not change. In addition, butanoyl-CoA was also detected as could be seen in Fig. 2. As for strain EL20, there was a 37-fold decrease in the concentration of acetyl-CoA (Fig. 2), and 5.3-fold decrease for malonyl-CoA (Fig. 2) compared to the wild type. In addition, EL20 showed 2.1-fold increase in butanoyl-CoA compared to EL14, (Fig. 2). These data signify that strains EL14 and EL20 consumed acetyl-CoA to form butanoyl-CoA. Our result indicates that strain EL20 also consumed malonyl-CoA to form butanoyl-CoA. Moreover, strain EL20 more effectively converted acetyl-CoA to butanoyl-CoA than strain EL14 (Fig. 2). Hence, the ATP-driven malonyl-CoA-mediated reaction (present in EL 20 and BUOH-SE) facilitated the consumption of acetyl-CoA for 1-butanol biosynthesis better than the direct condensation reaction. More importantly, we were able to validate our CoA profiling method since the genotypes of the strains can be explained based on their differences at the metabolite level.

### Reductive reaction of butanoyl-CoA to butanal is a possible rate-limiting step of 1-butanol production in cyanobacteria

NADPH is a more suitable driving force for the reductive reaction than NADH since the light reaction of photosynthesis produces NADPH, resulting in a more abundant amount of NADPH than NADH in the cyanobacterial cell (Tamoi et al. 2005). Oxygen-tolerant enzymes are also preferable than oxygen-sensitive enzymes for cyanobacterial 1-butanol production due to oxygen evolution via a photosynthetic activity. Specifically, substituting the NADPH-dependent enzyme for an NADH-dependent type in the reductive reaction of butanoyl-CoA to butanal played a key role in the improvement of 1-butanol productivity (Lan and Liao 2012). Furthermore, replacing the oxygen-sensitive enzyme with an oxygen-tolerant type dramatically increased 1-butanol productivity (Lan et al. 2013). In this study, strain EL20 possesses an oxygen-sensitive NADH-dependent aldehyde/alcochol dehydrogenase encoded by *adhE2*, EL22 has the oxygen-sensitive NAD(P)H-dependent butanal dehydrogenase encoded by *bldH* and BUOH-SE has the oxygen-tolerant NADH-dependent CoA-acylating propionaldehyde dehydrogenase encoded by *pduP* (Fig. 1; 1). We investigated the effect of each modification on the metabolic state of the pathway through quantitative metabolic profiling. Our result showed that the intracellular concentration of butanoyl-CoA in strain BUOH-SE was comparable to that of strains EL20 and EL22 and there was no significant difference in the intracellular butanoyl-CoA concentration between strains EL20, EL22 and BUOH-SE even though 1-butanol productivity of the strains was completely different (Table 1). These results indicate that there is still intracellular butanoyl-CoA available as a precursor for 1-butanol biosynthesis in spite of a series of improvements in the reductive reaction of butanoyl-CoA to butanal. Therefore, this suggests that butanoyl-CoA reduction is a possible limiting step for 1-butanol production. In this case, further enhancement of butanoyl-CoA reduction in cyanobacteria may be beneficial to increase 1-butanol productivity.

### Enhanced acetyl-CoA synthesis in strain BUOH-SE

**Fig. 3:**
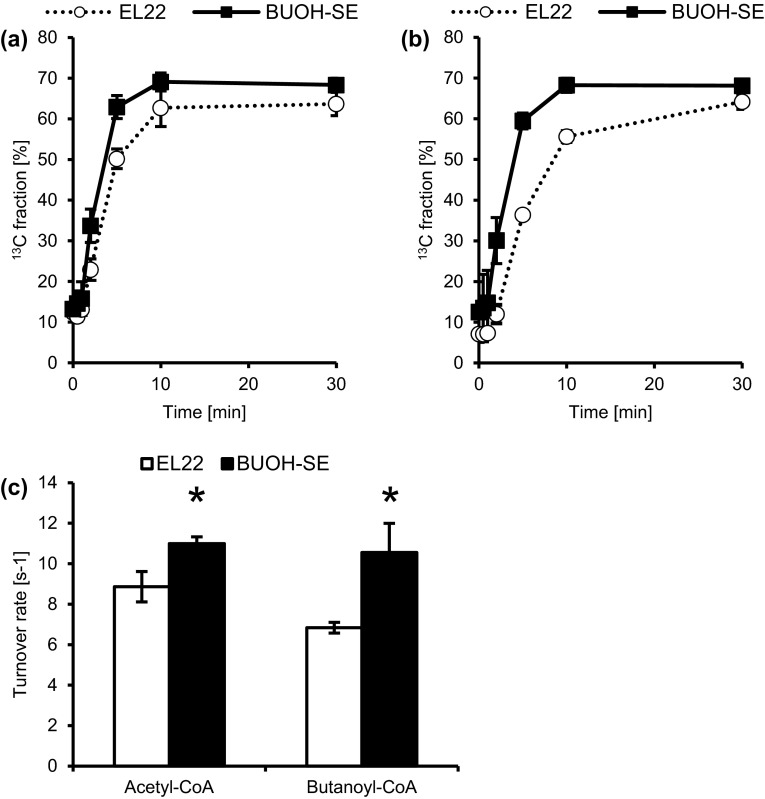
^13^C fraction change of acetyl-CoA (**a**) and butanoyl-CoA (**b**) with respect to time in strain EL22 and BUOH-SE. **c** Estimated metabolic turnover rate of acetyl-CoA and butanoyl-CoA in strain EL22 and BUOH-SE. The turnover rate was calculated on the basis of the initial slope of ^13^C fraction versus time plot. *Asterisk* (*) means a statistically-significant difference in turnover rate of the corresponding metabolite between the strains at *P* < 0.05 by means of Student’s *t* test. *Error bar* shows standard deviation

Intracellular acetyl-CoA and malonyl-CoA were depleted in strain EL22 due to the effective consumption of acetyl-CoA to butanoyl-CoA through malonyl-CoA as previously described (Fig. 2). However, there was an unexpected increase in the intracellular concentration of acetyl-CoA (9.8-fold) and malonyl-CoA (5.1-fold) in strain BUOH-SE compared to strain EL22 even though strain BUOH-SE also possesses an ATP-driven malonyl-CoA-mediated reaction (Fig. 2). Based on this observation, we hypothesized that the rate of acetyl-CoA and malonyl-CoA synthesis in strain BUOH-SE was increased compared to strain EL22 due to the effect of introducing the oxygen-tolerant enzyme PduP. In order to validate this hypothesis, we performed kinetic profiling of acetyl-CoA and butanoyl-CoA by means of metabolic turnover analysis using ^13^C-labelled sodium bicarbonate. Kinetic profiling of malonyl-CoA was not done since the intracellular malonyl-CoA concentration of strain EL22 was too low to perform metabolic turnover analysis. Figure 3a shows the ^13^C fraction change of acetyl-CoA with respect to time. The turnover rate of acetyl-CoA was estimated using the initial slope of the ^13^C fraction versus time curve (Fig. 3c) in which unlabeled acetyl-CoA in strain BUOH-SE was replaced with ^13^C-labelled acetyl-CoA faster than in strain EL22 (at time point between 2 and 5 min). The turnover rate of acetyl-CoA in strain BUOH-SE was higher than in strain EL22 (Fig. 3a, c). In this case, the intracellular concentration of acetyl-CoA was higher in strain BUOH-SE than in strain EL22 (Fig. 2). Since the rate of ^13^C-labeling depends on the pool size of each metabolite in the cell (Hasunuma et al. 2010), a given metabolite with higher intracellular concentration and turnover rate will have a higher carbon incorporation rate. Hence, the carbon incorporation rate of acetyl-CoA in strain BUOH-SE was increased compared to that of strain EL22 indicating that the rate of acetyl-CoA synthesis in strain BUOH-SE was higher than in strain EL22.

In case of butanoyl-CoA, a trend in the kinetic profiling similar to acetyl-CoA was also observed in which unlabeled butanoyl-CoA in strain BUOH-SE was replaced with ^13^C-labelled butanoyl-CoA faster than in strain EL22 (at time point between 2 and 10 min). The turnover rate of butanoyl-CoA in strain BUOH-SE was also higher than in strain EL22 (Fig. 3b, c). Here, there was no significant difference in the intracellular concentration of butanoyl-CoA between strain EL22 and BUOH-SE (Fig. 2). Therefore, the increased carbon incorporation rate of butanoyl-CoA in strain BUOH-SE compared to strain EL22 indicated that the rate of butanoyl-CoA synthesis in strain BUOH-SE was higher than in strain EL22.

### Investigation of the factors that affect the increased rate of acetyl-CoA synthesis in strain BUOH-SE

Next, we investigated the factors that contributed to the increased rate of acetyl-CoA synthesis in strain BUOH-SE. Pyruvate is a main precursor for acetyl-CoA biosynthesis through the central metabolism, thus the increased carbon incorporation rate of acetyl-CoA is expected to correlate with increased pyruvate consumption. Under the assumption that the effective regeneration of NAD^+^ via the reductive reaction catalyzed by PduP enabled the increased supply of NAD^+^ for the conversion of pyruvate to acetyl-CoA catalyzed by pyruvate dehydrogenase complex (PDH), we initially hypothesized that the insufficient NAD^+^ pool was the limiting factor of strain EL22 for converting pyruvate to acetyl-CoA via PDH. However, our result showed that the intracellular NAD^+^ concentration of strain EL22 was slightly higher than that of strain BUOH-SE (Table 2), which meant that the intracellular NAD^+^ concentration of strain EL22 is enough to convert pyruvate to acetyl-CoA via PDH and is therefore not the limiting factor of EL22.

**Table 2:** Intracellular concentration of NAD^+^ and NADH and intracellular NADH/NAD^+^ ratio in strain EL22 and BUOH-SE

	NAD^+^ (pmol/mg-dcw)	NADH (pmol/mg-dcw)	NADH/NAD^+^ ratio
EL22	161 ± 23	44 ± 20	0.29 ± 0.13
BUOH-SE	135 ± 13	51 ± 16	0.39 ± 0.14

The values are the mean ± SD of three replicates

**Fig. 4:**
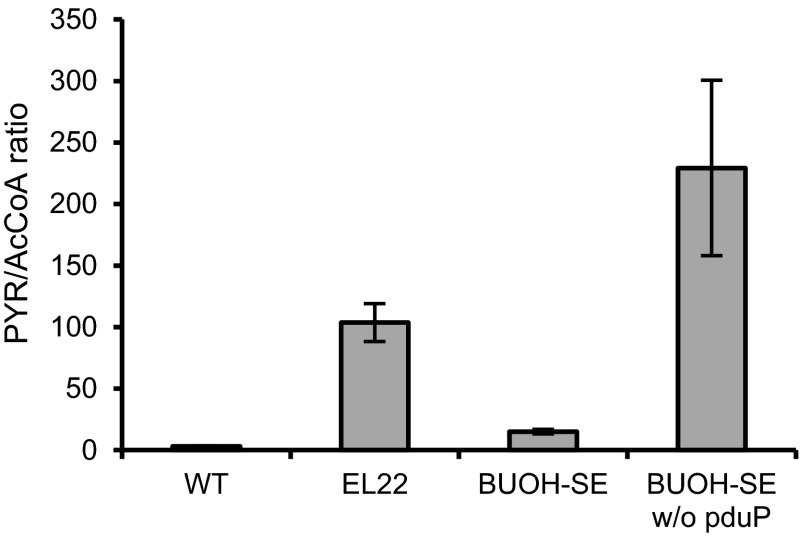
PYR/AcCoA ratio in wild type, strain EL22, BUOH-SE w/o *pduP* and BUOH-SE. *Error bar* shows standard deviation

Thus, we focused on free CoA, the co-factor that is necessary for the conversion of pyruvate to acetyl-CoA. We assumed that a more effective regeneration of free CoA from butanoyl-CoA via the reductive reaction catalyzed by PduP enabled the adequate supply of free CoA for the conversion of pyruvate to acetyl-CoA. Since free CoA is undetectable due to its low intracellular concentration, we constructed a strain BUOH-SE w/o *pduP* incapable of regenerating free CoA from butanoyl-CoA to investigate how free CoA that was regenerated from butanoyl-CoA affected the conversion of pyruvate to acetyl-CoA. In this strain, *ter*, *nphT7*, *phaJ*, and *phaB* genes were expressed but not PduP (Table 1) thus, it was identical to strain EL22 or BUOH-SE except for its ability to produce 1-butanol from butanoyl-CoA through butanal. Figure 4 shows the ratio of intracellular concentration of pyruvate to acetyl-CoA (PYR/AcCoA ratio) in wild type, strains EL22, BUOH-SE w/o *pduP*, and BUOH-SE. PYR/AcCoA ratio of strain BUOH-SE markedly decreased compared to that of strain EL22, which indicated that the conversion of pyruvate to acetyl-CoA was enhanced in strain BUOH-SE compared to strain EL22. In contrast, strain BUOH-SE w/o *pduP* showed a higher PYR/AcCoA ratio than EL22 (Fig. 4), which strongly suggests that free CoA has to be regenerated from butanoyl-CoA for the conversion of pyruvate to acetyl-CoA. In case of strain BUOH-SE, the introduction of PduP gene unexpectedly enhanced free CoA recycling, resulting to an increase in 1-butanol production.

## Discussion

In this study, we characterized the differences in 1-butanol biosynthesis of strains with different genetic backgrounds by quantitative metabolic profiling of acyl-CoAs. Acetoacetyl-CoA, 3-hydroxybutyryl-CoA and crotonoyl-CoA were undetectable in all strains, and as well as their corresponding ^13^C-labelled forms in the ^13^C-labelled cell extract. Considering the fact that there was no significant loss of the metabolites during the extraction procedure (Table S3), we inferred that the intracellular concentration of these three acyl-CoAs was too low compared to the detectable levels of other acyl-CoAs namely acetyl-CoA, malonyl-CoA and butanoyl-CoA. We therefore inferred that there was no bottleneck reaction in the conversion of acetoacetyl-CoA to crotonoyl-CoA through 3-hydroxybutyryl-CoA. In fact, acetyl-CoA was effectively converted to butanoyl-CoA in both NADH-dependent reaction (in strain EL14 and strain EL20) and NADPH-dependent reaction (in strain EL22 and strain BUOH-SE) (Figs. 1, 2). In the case of low expression levels of (*S*)-3-hydroxybutyryl-CoA dehydrogenase encoded by *hbd*, the conversion of acetoacetyl-CoA to (*S*)-3-hydroxybutyryl-CoA becomes a rate-limiting step in the production of butanoyl-CoA (Fischer et al. 2010). Therefore, the expression levels of (*S*)-3-hydroxybutyryl-CoA dehydrogenase in the 1-butanol-producing cyanobacteria may be enough to facilitate the conversion of acetoacetyl-CoA to (*S*)-3-hydroxybutyryl-CoA. Regarding the conversion of crotonoyl-CoA to butanoyl-CoA, it is catalyzed by the NADH-dependent trans-enoyl-CoA reductase Ter derived from *Treponema denticola* in the engineered cyanobacteria. This enzyme is oxygen-tolerant and irreversible compared to the native enzyme in *Clostridium* species catalyzing the conversion of crotonoyl-CoA to butanoyl-CoA, which is oxygen-sensitive and reversible (Inui et al. 2008). Hence, it is possible that there was a more straightforward conversion of crotonoyl-CoA to butanoyl-CoA in the engineered cyanobacteria. Using quantitative target analysis of acyl-CoAs in the 1-butanol biosynthesis, we validated our previous hypothesis that the oxygen sensitivity of CoA-acylating aldehyde dehydrogenase is a possible key limiting factor in cyanobacteria for the production of 1-butanol through the CoA-dependent pathway (Lan et al. 2013). In this study, we also showed that intracellular butanoyl-CoA is still available as a precursor for 1-butanol biosynthesis. Therefore, improving the enzyme activity of CoA-acylating aldehyde dehydrogenase or substituting another enzyme with more preferable kinetic parameters such as *K*_m_ and *k*_cat_ may further improve the 1-butanol productivity. Another presumable strategy is to utilize acetate CoA-transferase which catalyzes the reaction of butyrate and acetyl-CoA production from butanoyl-CoA and acetate (Vanderwinkel et al. 1968). Subsequently, butyrate can be converted to 1-butanol via two reactions catalyzed by carboxylic acid reductase and aldehyde dehydrogenase, respectively (Pásztor et al. 2014). This additional bypassing pathway may play a role to increase consumption rate of butanoyl-CoA for 1-butanol biosynthesis.

Using metabolic turnover analysis, kinetic profiling also revealed the unexpected effect of introducing the oxygen-tolerant PduP for enhancing acetyl-CoA synthesis. Considering the higher PYR/AcCoA ratio in strain EL22 compared to wild type (Fig. 4), the conversion of pyruvate to acetyl-CoA is a possible bottleneck step in strain EL22 for 1-butanol biosynthesis through the CoA-dependent pathway. The increased rate of acetyl-CoA synthesis and the decreased PYR/AcCoA ratio in strain BUOH-SE compared to strain EL22, however, indicate that strain BUOH-SE overcame the bottleneck step. Intracellular NAD^+^ and NADH pools were comparable in strain EL22 and BUOH-SE (Table 2), thus the redox co-factor regeneration was not a limiting factor for the conversion of pyruvate to acetyl-CoA via PDH. Strain BUOH-SE w/o *pduP* is incapable of regenerating free-CoA from butanoyl-CoA because it lacks CoA-acylating aldehyde dehydrogenase. Since the PYR/AcCoA ratio of the strain BUOH-SE w/o *pduP* was also high similar with strain EL22 (Fig. 4), we therefore infer that regeneration of free CoA from butanoyl-CoA is a key factor for the conversion of pyruvate to acetyl-CoA. It has been reported that in vitro synthetic system for the poly (3-hydroxybutyric acid) formation can utilize free CoA recycling (Jossek and Steinbüchel 1998) and repeated recycling of free CoA in rat liver mitochondria occurs (Osmundsen and Sherratt 1975). Therefore, it is reasonable to assume that free CoA released from butanoyl-CoA is recycled for the conversion of pyruvate to acetyl-CoA in the cyanobacteria. We expected that butanoyl-CoA would accumulate in strain BUOH-SE w/o *pduP* more than strain EL22 and BUOH-SE, however, the intracellular concentration was comparable among the strains (Fig. 4). Moreover, the sum of the intracellular concentration of acetyl-CoA, malonyl-CoA and butanoyl-CoA was also comparable among the strains (Fig. 4). These results indicate that the amount of the intracellular acyl-CoAs available in the CoA-dependent 1-butanol biosynthesis pathway has an upper limit. This fact also indicates the importance of regeneration of free-CoA from butanoyl-CoA for the further synthesis of acetyl-CoA from pyruvate. Considering such importance of regeneration of free CoA from butanoyl-CoA for increasing the rate of acetyl-CoA synthesis, the reduction of butanoyl-CoA to butanal must be enhanced to improve the recycling efficiency of free-CoA.

## Conclusion

We investigated in vivo the metabolic state in the CoA-dependent 1-butanol biosynthesis pathway of cyanobacteria by quantitative target analysis and kinetic profiling of acyl-CoAs in the CoA-dependent pathway through (RP-IP-LC)/QqQ-MS. The quantification of intracellular concentration of acyl-CoAs in the 1-butanol biosynthesis pathway validated at the metabolite level that the ATP-driven malonyl-CoA-mediated reaction effectively consumed acetyl-CoA for butanoyl-CoA synthesis as intended and indicated that the reductive reaction of butanoyl-CoA to butanal was a possible rate-limiting step in the cyanobacterial 1-butanol biosynthesis through the CoA-dependent pathway. Moreover, the kinetic profiling of acetyl-CoA and butanoyl-CoA using metabolic turnover analysis revealed the unexpected increase of acetyl-CoA synthesis rate in strain BUOH-SE. We inferred that the effective regeneration of free-CoA from butanoyl-CoA played an important role in enhancing the conversion of pyruvate to acetyl-CoA. These facts strongly indicate that the reductive reaction of butanoyl-CoA to butanal should be additionally modified to improve 1-butanol productivity in the cyanobacteria. The information shown in this study should be helpful for further engineering of cyanobacteria for improved 1-butanol production.

## Electronic supplementary material

Supplementary material 1 (DOCX 24 kb)
