# Supplementary material for: Quantitative target analysis and kinetic profiling of acyl-CoAs reveal the rate-limiting step in cyanobacterial 1-butanol production
Source: Metabolomics. 2016 Jan 4;12:26. doi: 10.1007/s11306-015-0940-2 (PMC4700068; doi:10.1007/s11306-015-0940-2)
Supplement: Supplementary file 1 — Supplementary material 1 (DOCX 24 kb) [file 11306_2015_940_MOESM1_ESM.docx]

**Supporting information**

**Table S1**. Calibration curves of targeted metabolites obtained by (RP-IP-LC)/QqQ-MS. x is the area ratio of monoisotopic peak to uniformly ^13^C-labeled peak and y is naturally labeled standard amount in pmol per tube. U-^13^C / (U-^13^C+U-^12^C) means the ratio of U-^13^C to (U-^13^C + U-^12^C) peak area in internal standard

| Metabolite | Range [pmol/tube] | Equation | R^2^ | U-^13^C / (U-^13^C+U-^12^C) |
| --- | --- | --- | --- | --- |
| Pyruvate | 313 ~ 8460 | y = 34.161x + 150.07 | 0.997 | 0.98 |
| Acetyl-CoA | 1 ~ 243 | y = 73.502x + 0.4931 | 0.999 | 1.00 |
| Malonyl-CoA | 0.25 ~ 16 | y = 0.19x + 0.3414 | 0.996 | 1.00 |
| Butanoyl-CoA | 1 ~ 243 | y = 2.525x + 2.9743 | 0.997 | 0.99 |

**Table S2**. Analytical parameters for (RP-IP-LC)/QqQ-MS analysis. Single dagger (^†^) means MRM transitions for the analysis of U-^12^C metabolite and double daggers (^††^) mean MRM transitions for the analysis of U-^13^C metabolite. MRM transitions with single dagger and no mark were used for metabolic turnover analysis of acetyl-CoA and butyryl-CoA. *1 RT, Retention time *2 DT, Dwell time *3 Q1PB, Q1 pre bias *4 CE, Collision energy *5 Q3 PB, Q3 pre bias

|  | Metabolite | RT*^1^  [min] | MRM transitions | DT*^2^  [msec] | Q1 PB*^3^ [V] | CE*^4^  [V] | Q3 PB*^5^ [V] |
| --- | --- | --- | --- | --- | --- | --- | --- |
| Run 1 | Pyruvate | 8.489 | ^†^147.05 > 87, ^††^150.05 > 90 | 30 | 24 | 9 | 15 |
| Run 2 | Acetyl-CoA | 7.563 | ^†^808.1 > 408.1, 809.1 > 408.1  810.1 > 408.1, ^††^831.1 > 418.1 | 45 | 20 | 37 | 28 |
|  | Malonyl-CoA | 7.732 | ^†^852.1 > 408.1, ^††^876.1 >418.1 | 50 | 32 | 45 | 26 |
|  | Butanoyl-CoA | 10.623 | ^†^836.1 > 408.1, 837.1 > 408.1  838.1 > 408.1, 839.1 > 408.1  ^††^861.1 >418.1 | 45 | 31 | 37 | 26 |

**Table S3**. Recovery rate in the extraction procedure of acyl-CoAs. Recovery rate was calculated by dividing the area of each acyl-CoA in the standard mixture by the area of the corresponding acyl-CoA through the extraction procedure. Values shown are the average of duplicate experiments. Abbreviations as follows: Ac-CoA, Acetyl-CoA; AcAc-CoA, Acetoacetyl-CoA; Mal-CoA, Malonyl-CoA; 3-HB-CoA, 3-Hydroxybutyryl-CoA; Cr-CoA, Crotonoyl-CoA; Bu-CoA, Butanoyl-CoA

|  | Ac-CoA | Free CoA | AcAc-CoA | Mal-CoA | 3-HB-CoA | Cr-CoA | Bu-CoA |
| --- | --- | --- | --- | --- | --- | --- | --- |
| Recovery rate [%] | 58 | 63 | 65 | 59 | 59 | 59 | 67 |
